# Suppression of Osteosarcoma Cell Invasion by Chemotherapy Is Mediated by Urokinase Plasminogen Activator Activity via Up-Regulation of EGR1

**DOI:** 10.1371/journal.pone.0016234

**Published:** 2011-01-20

**Authors:** Yukihiro Matsunoshita, Kosei Ijiri, Yasuhiro Ishidou, Satoshi Nagano, Takuya Yamamoto, Hiroko Nagao, Setsuro Komiya, Takao Setoguchi

**Affiliations:** 1 Department of Orthopaedic Surgery, Graduate School of Medical and Dental Sciences, Kagoshima University, Kagoshima, Japan; 2 Department of Medical Joint Materials, Graduate School of Medical and Dental Sciences, Kagoshima University, Kagoshima, Japan; Queensland University of Technology, Australia

## Abstract

**Background:**

The cellular and molecular mechanisms of tumour response following chemotherapy are largely unknown. We found that low dose anti-tumour agents up-regulate *early growth response 1* (*EGR1*) expression. EGR1 is a member of the immediate-early gene group of transcription factors which modulate transcription of multiple genes involved in cell proliferation, differentiation, and development. It has been reported that EGR1 act as either tumour promoting factor or suppressor. We therefore examined the expression and function of *EGR1* in osteosarcoma.

**Methods:**

We investigated the expression of EGR1 in human osteosarcoma cell lines and biopsy specimens. We next examined the expression of EGR1 following anti-tumour agents treatment. To examine the function of EGR1 in osteosarcoma, we assessed the tumour growth and invasion in vitro and in vivo.

**Results:**

Real-time PCR revealed that *EGR1* was down-regulated both in osteosarcoma cell lines and osteosarcoma patients' biopsy specimens. In addition, EGR1 was up-regulated both in osteosarcoma patient' specimens and osteosarcoma cell lines following anti-tumour agent treatment. Although forced expression of EGR1 did not prevent osteosarcoma growth, forced expression of EGR1 prevented osteosarcoma cell invasion in vitro. In addition, forced expression of EGR1 promoted down-regulation of urokinase plasminogen activator, urokinase receptor, and urokinase plasminogen activity. Xenograft mice models showed that forced expression of EGR1 prevents osteosarcoma cell migration into blood vessels.

**Conclusions:**

These findings suggest that although chemotherapy could not prevent osteosarcoma growth in chemotherapy-resistant patients, it did prevent osteosarcoma cell invasion by down-regulation of urokinase plasminogen activity via up-regulation of EGR1 during chemotherapy periods.

## Introduction

Osteosarcoma is the most frequent primary malignant bone tumor. After initial diagnosis is made by biopsy, treatment consists of preoperative chemotherapy, followed by definitive surgery and postoperative chemotherapy. The Survival rates for patients treated with intensive multidrug chemotherapy and aggressive local control have been reported at 60–80% [1]–[5]. Indeed, patients with non-metastatic disease have a 70% chance of long-term survival. Eighty percent of patients die of metastatic disease, most commonly in the lungs [3]. Unfortunately, patients with metastatic disease at diagnosis or those who have recurrent disease have a poor prognosis, with only 20% surviving at 5 years, indicating that new therapeutic options for them need to be actively explored [6], [7].

The early growth response gene 1 (*EGR1*) is a member of the immediate-early gene group of transcription factors which modulate transcription of multiple genes involved in cell proliferation, differentiation, and development [8]. Expression of *EGR1* is significantly reduced in a number of tumor cells [9], [10], and loss of expression of it is closely associated with tumor formation in mammalian cells and tissues [10]. On the other hand, stable expression of *EGR1* inhibited cell proliferation and soft agar growth in NIH3T3 cells transformed with v-*sis*, indicating that *EGR1* functions as a tumor suppressor [11]. We therefore examined the expression and function of *EGR1* in osteosarcoma. Here, we report that expression of *EGR1* is down-regulated in human osteosarcoma cell lines and patient’ biopsy specimens. In addition, treatment with anti-tumour agents promoted up-regulation of *EGR1*. Although forced expression of EGR1 did not affect osteosarcoma growth, forced expression of EGR1 inhibited osteosarcoma cell invasion by down-regulation of urokinase plasminogen activator (uPA) and urokinase receptor (uPAR).

## Materials and Methods

### Cell culture

143B, Saos-2, HOS, and MG63 cells were purchased from the American Type Culture Collection (ATCC, USA). NOS-1 was provided by the RIKEN BRC through the National Bio-Resource Project of The MEXT, Japan (Tsukuba, Japan) [12]. Cells were grown in Dulbecco's modified Eagle's medium (DMEM) supplemented with 10% FBS, penicillin (100 U/ml), and streptomycin (100 µg/ml). Human osteoblast cells (NHOst) were purchased from Sanko Junyaku (Tokyo, Japan). NHOst was cultured with OBM™ (Cambrex, East Rutherford, NJ, USA) or DMEM supplemented with 10% FBS. All cells were grown in a humidified atmosphere containing 5% CO_2_ at 37°C.

### Anti-tumor agents

Doxorubicin, methotrexate, and etoposide were purchased from Sigma-Aldrich (MO, USA). Cisplatin was purchased from LKT laboratories (MN, USA).

### Patient’ specimens

All human osteosarcoma biopsy specimens were obtained from primary lesions. Biopsy was performed before chemotherapy or radio therapy to make the diagnosis. Normal bone tissue was obtained from femur during total hip arthroplasty. Specimens of OS6, OS8, and OS9 tumors were obtained during tumor resection in osteosarcoma patients who received chemotherapy. Doxrubicin, methotrexate, and cisplatin were given to these three patients according to COSS-86 protocol. We compared the EGR1 expressions in the biopsy specimens and the resected tumor specimens obtained from these patients. The study protocol was approved by the institutional review board of the Kagoshima University. All patients and controls gave written informed consent.

### Real-time PCR

For real-time PCR, total RNA was obtained 24 h, 48 h, and 5 days following drug treatment. DNase-treated and reverse-transcribed using oligo(dT) primers as described by the manufacturer (Invitrogen, Carlsbad, CA, USA). Reactions were run using SYBR Green (BIO-RAD, Hercules, CA, USA) on a MiniOpticon™ machine (BIO-RAD). The comparative Ct (ΔΔCt) method was used to determine fold change in expression using *GAPDH* or *ACTB*. Each sample was run minimally at three concentrations in triplicate. All primer sets amplified 150- to 200-bp fragments. The primers sequences used were follows: for *EGR1*: 5-CAGCACCTTCAACCCTCAG-3, 5- CACAAGGTGTTGCCACTGTT-3; *uPA*: 5- TGTGAGATCACTGGCTTTGG-3, 5- GTCAGCAGCACACAGCATTT-3; *uPAR*: 5- TGAAGAACAGTGCCTGGATG-3, 5- TGTTGCAGCATTTCAGGAAG-3; *GAPDH*: 5- GAAGGTGAAGGTCGGAGTC-3, 5- GAAGATGGTGATGGGATTTC-3; *ACTB*: 5-AGAAAATCTGGCACCACACC-3, 5-AGAGGCGTACAGGGATAGCA-3.

### MTT assay

Following 100 ng–1 µg cisplatin, 1 ng–10 ng methotrexate, 50 ng–1000 ng etoposide, or 10 ng–100 ng doxorubicin treatment, we performed MTT assay to evaluate the osteosarcoma growth as previously reported [13]. In addition, we transfected control vector or EGR1 expression vector, and examined osteosarcoma cell growth by MTT assay. Cells were incubated with substrate for MTT (3-(4,5-dimethylthiazol-2-yl)-2,5-diphenyltetrazolium bromide) for 4 hours, and washed with PBS and lysed to release formazan from cells. Then cells were analyzed in a Safire microplate reader (BIO-RAD) at 562 nm.

### Vector transfection

EGR1 expression vector was purchased from Origene (Maryland, USA). EGR1 was cloned into pCMV6-Entry Neomycin Vector. Lipofection of expression vector was performed as recommended in the supplier's protocol using FuGENE 6 (Roche, Basel, Switzerland). All transfected cells were treated with neomycin constitutively to obtain stable transfectants. EGR stable transfectants were used for invasion assay, examinations of uPA and uPAR expressions, and in vivo experiments.

### Colony formation assay

Colony formation assay was performed as previously described [14]. Briefly, cells were suspended in DMEM containing 0.33% agar and 10% fetal bovine serum and plated onto the bottom layer containing 0.5% agar. The cells were plated at a density of 5×10^3^ per well in a 24-well plate, and colonies were counted 14 days later. Each condition was analyzed in triplicate, and all experiments were repeated three times.

### Invasion assay

Invasion of osteosarcoma cells was measured using the BD BioCoat™ BD Matrigel™ Invasion Chamber (BD Bioscience, NJ, U.S.A.) according to the manufacturer's protocol. Briefly, the cells were transfected with plasmids and selected by neomycin. Osteosarcoma cells were seeded onto the membrane of the upper chamber of the transwell at a concentration of 3–5×10^5^/ml in 2 ml of DMEM medium. The medium in the upper chamber was serum-free. The medium in the lower chamber contained 5% fetal calf serum as a source of chemoattractants. Cells that passed through the Matrigel-coated membrane were stained with Diff-Quik (Sysmex, Kobe, Japan) and photographed.

### Western blot

Western blot analysis was performed as previously reported [15]. Briefly, cells were lysed using NP40 lysis buffer (0.5% NP40, 10 mM Tris-HCl (pH 7.4), 150 mM NaCl, 3 mM pAPMSF (Wako Chemicals, Kanagawa, Japan), 5 mg/ml aprotinin (Sigma, StLouis, USA), 2 mM sodium orthovanadate (Wako Chemicals, Kanagawa, Japan), and 5 mM EDTA). Lysates were subjected to SDS-PAGE and subsequent immunoblotting was performed. Following antibodies were used: ant- EGR1 and anti-beta actin (Santa Cruz, CA. U.S.A). Detection was performed using the ECL detection system (Amersham, Giles, UK).

### uPA activity assay

uPA activity assay was performed with cell extracts according to the manufacturer's instructions, with absorption measured at 340 nm (Innovative Research, MI, U.S.A.). The assay measures only the active species of uPA, and a standard curve was generated using recombinant active uPA. The assay conditions were optimized so that the amount of tissue extract or cell extract added gave rise to uPA activity within the linear range of detection. Each reaction was performed in triplicate, and all experiments were repeated for three times.

### Xenograft model of osteosarcoma

For subcutaneous xenograft models, 143B cells were suspended in 100 µL Matrigel (BD, NJ USA.). Cell suspensions were subcutaneously inoculated in nude mice. Three weeks after inoculation, 4 µg/kg doxorubicin was administered by intraperitoneal injection. One day after treatment, mice were scarified and tumors were examined. For metastasis experiments, 143B cells (5×10^5^) were transfected with GFP lentiviral particles (Santa Cruz, CA. U.S.A). Stably-GFP-expressing 143B cells (1×10^6^) were mixed with a collagen gel in a 1∶1 volume, and inoculated into the left knee joint of 6-weeks-old nude mice. Five weeks after inoculation, the mice were sacrificed. GFP-positive-143B cells were counted in 50 µl blood aspirates from hearts using the M165 FC microscope (Leica Microsystems, Wild Heerbrugg, Switzerland). Metastatic nodules in the lungs were evaluated by direct microscopic visualization using an M165 FC microscope. Lung metastasis area was calculated by Lumina Vision (Mitani Corporation, Tokyo, Japan). All experimental procedures were performed in compliance with the guiding principles for the Care and Use of Animals described in the American Journal of Physiology and with the Guidelines established by the Institute of Laboratory Animal Sciences, Faculty of Medicine, Kagoshima University (approval number: 20064). All efforts were made to minimize animal suffering, to reduce the number of animals used, and to utilize possible alternatives to in vivo techniques.

### ELISA

Expression levels of uPA and uPAR proteins were assayed using specific enzyme-linked immunosorbent assay kits according to the manufacturer's instructions (Abnova, Taipei, Taiwan). Cell lysates were collected by EGR1 stable transfected osteosarcoma cells.

### Statistics

Each sample was analyzed in triplicate, and experiments were repeated three times. In all figures, error bars are standard deviations. All statistical analyses were performed using Microsoft Office Excel (Microsoft, Albuquerque, New Mexico, USA) and STASTISCA (StatSoft, Tulsa, OK, USA). Differences between mean values were evaluated by the unpaired *t*-test, and differences in frequencies by Fisher's exact test. Differences were considered significant at *P*<0.05.

## Results

### EGR1 is down-regulated in osteosarcoma cell lines and patient’ specimens

**Figure 1:**
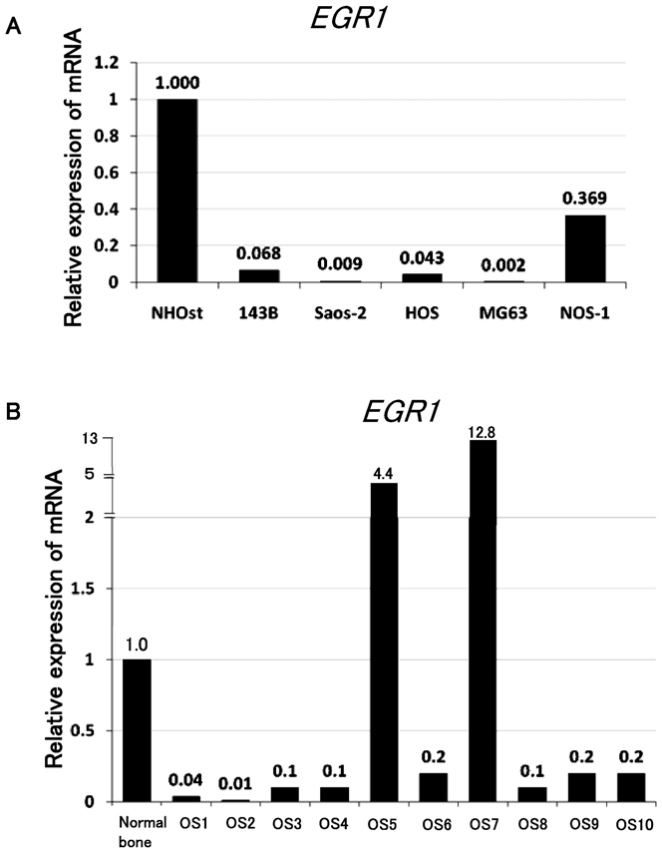
Down-regulation of *EGR1* in human osteosarcoma. Total RNA extracted from osteosarcoma cell lines (A) and osteosarcoma patients' biopsy specimens (B) were analyzed by real-time PCR. Results revealed that 5 of 5 human osteosarcoma cell lines and 8 of 10 human biopsy specimens of osteosarcoma had decreased *EGR1* expression. The comparative Ct (ΔΔCt) method was used to determine fold change in expression using *GAPDH*. These experiments were performed in triplicate with similar results.

Real-time PCR was performed to examine the gene expression of *EGR1* in osteoblast and osteosarcoma cell lines including NHOst, 143B, Saos-2, HOS, MG63, and NOS-1. Real-time PCR revealed that the 5 of 5 osteosarcoma cell lines exhibited 0.002- to 0.369-fold decreased in expression of *EGR1* (**Figure 1A**). In addition, we performed real-time PCR using patient’ biopsy specimens. Real-time PCR revealed that *EGR1* was decreased 0.01-to 0.2-fold in 8 of 10 human biopsy specimens (**Figure 1B**). These findings suggest that the *EGR1* is down-regulated in human osteosarcomas.

### Anti-tumour agent treatment promoted up-regulation of *EGR1*

**Figure 2:**
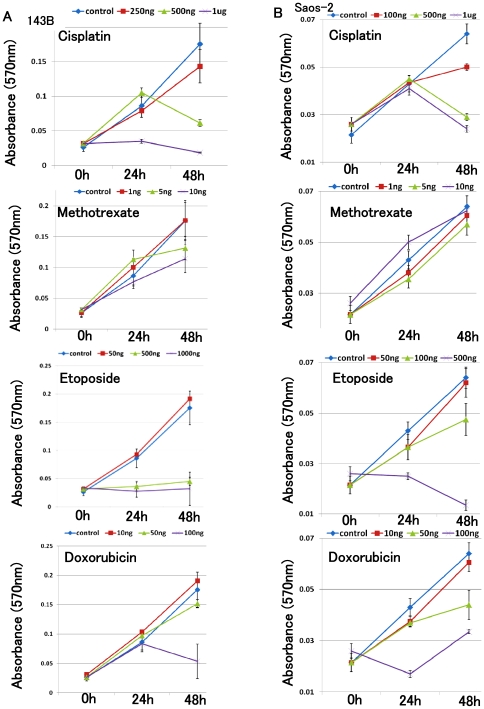
Osteosarcoma cell growth following anti-tumor drug treatment. MTT assay showed that growth at 48 h of 143B cells was not inhibited by 250 ng cisplatin, 1 ng/ml methotrexate, 50 ng/ml etoposide, or 10 ng/ml doxorubicin. Growth of 143B cell was inhibited by higher dose of each drug (*A*) (P<0.05). Growth at 48 h of Saos-2 cells was not inhibited by 1 ng/ml methotrexate, 5 ng/ml methotrexate, 10 ng/ml methotrexate, 50 ng/ml etoposide, or 10 ng/ml doxorubicin. Growth of Saos-2 cell was inhibited by higher dose of each drug (*B*) (P<0.05). The experiment was performed in triplicate with similar results [error bars represent mean (SD)].

**Figure 3:**
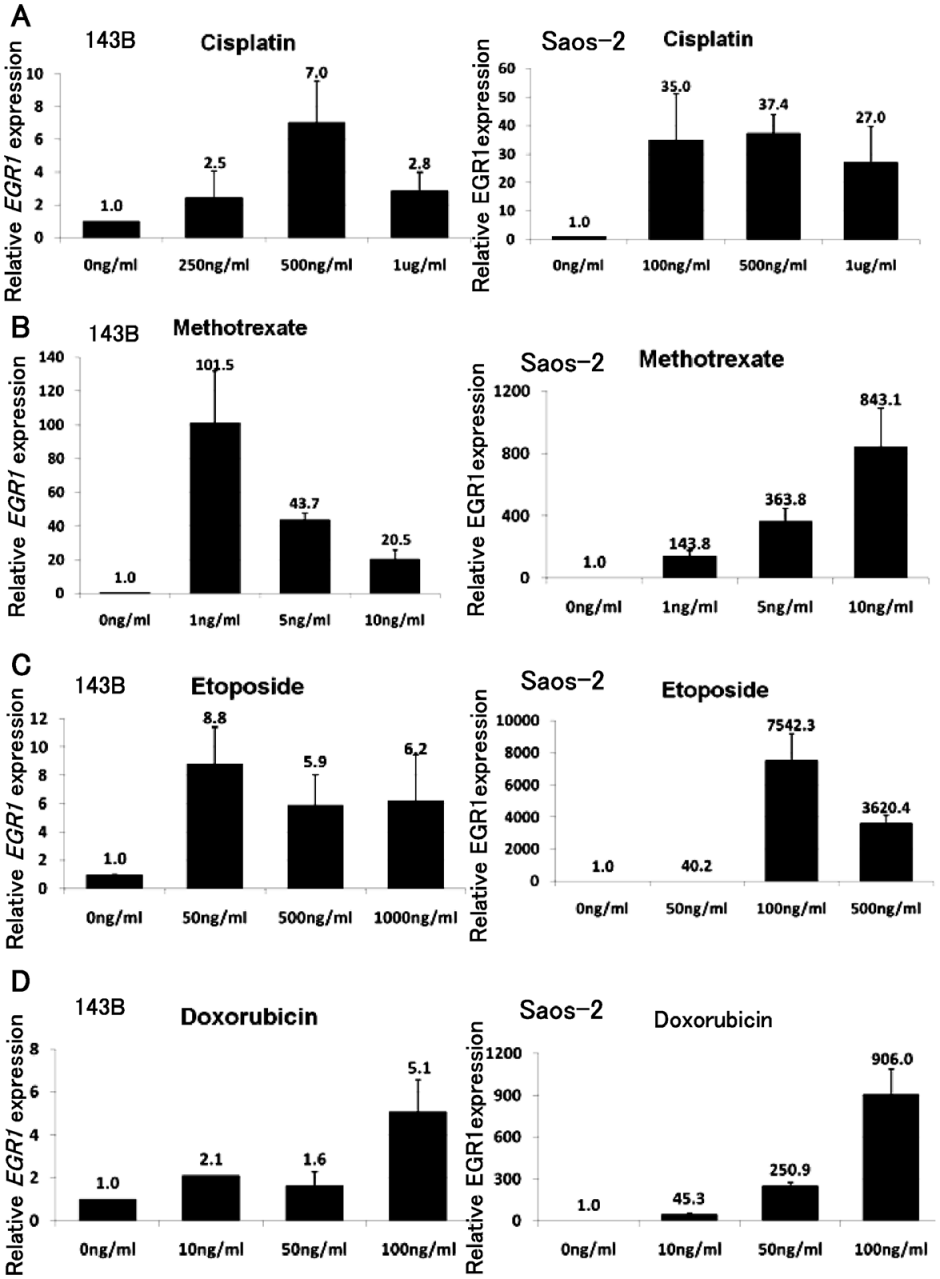
Anti-tumor agent treatment increased the expression of *EGR1*. Following 24 h drug treatments, total RNA extracted from osteosarcoma cell lines were analyzed by real-time PCR. Treatment with cisplatin, methotrexate, etoposide or doxorubicin increased *EGR1* expression in 143B and Saos-2 cells. The comparative Ct (ΔΔCt) method was used to determine fold change in expression using *GAPDH* or *ACTB*. Experiments were performed in triplicate with similar results [error bars represent mean (SD)].

To examine the effects of anti-tumour agents on *EGR1* expression, we performed real-time PCR after anti-tumour agent treatment. We attempt to clarify the changes in EGR1 expression following low-dose anti-tumor agent treatment, and determined anti-tumor drug concentrations required to prevent osteosarcoma cell proliferation. MTT assay revealed that 250 ng/ml cisplatin, 1 ng/ml methotrexate, 50 ng/ml etoposide, or 10 ng/ml doxorubicin treatment did not prevent 143B cell growth. Growth of Saos-2 cells was not inhibited by 1 ng/ml methotrexate, 5 ng/ml methotrexate, 10 ng/ml methotrexate, 50 ng/ml etoposide, or 10 ng/ml doxorubicin. On the other hand, Growth of 143B cell and Saos-2 cell was inhibited by higher dose of each drug (**Figure 2** **A, B**). Following 24 h treatment with these concentrations of anti-tumor drugs, *EGR1* was up-regulated (**Figure 3** **A–D**). Following 48 h or 5 days treatment, cisplatin, methotrexate, etoposide or doxorubicin increased *EGR1* expression in 143B cell and Saos-2 cells (**Figure S1**). We next examined the expression of *EGR1* following chemotherapy in biopsy specimens. Specimens of OS6, OS8, and OS9 tumors were obtained during tumor resection in osteosarcoma patients who received chemotherapy. We compared the *EGR1* expressions in the biopsy specimens and the resected tumor specimens obtained from these patients. In 3 of 3 patient’ specimens examined, *EGR1* expression was increased 7.87- to 1.71 following chemotherapy (**Figure S2A**). To examine the expression of *EGR1* following low-dose chemotherapy in vivo, we used a novel osteosarcoma murine xenograft model with 143B cells. We injected 4 µg/kg doxorubicin which is less than one-hundred dose of COSS-86 protocol for osteosarcoma patients. Real-time PCR showed that low dose doxorubicin treatment promoted up-regulation of *EGR1* in vivo (**Figure S2B**).

### Over-expression of EGR1 does not prevent osteosarcoma growth

**Figure 4:**
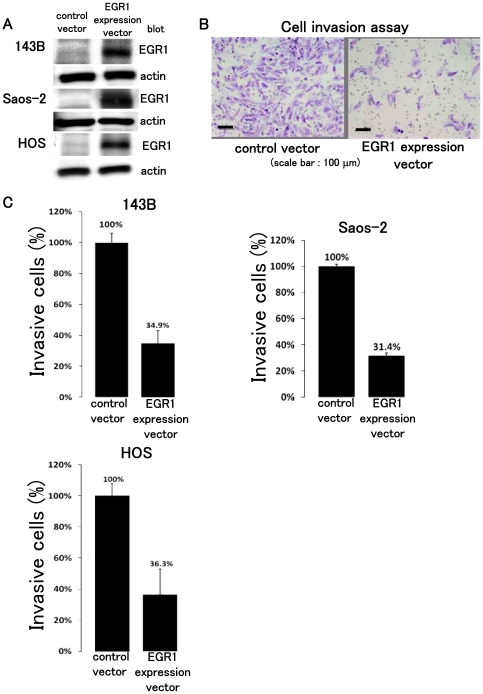
EGR1 prevents osteosarcoma cell invasion in vitro. Western blot analysis revealed that lysates of EGR1 expression vector-transfected cells were positive for anti-EGR1 antibody (*A*). Cell invasion assay showed that forced expression of EGR1 decreased 143B, Saos-2, and HOS cell invasion (*B*) (P<0.05). These experiments were performed in triplicate with similar results [error bars represent mean (SD)].

It has been reported that EGR1 over-expression suppresses the growth of cell in soft agar and tumor growth in nude mice [10], [16]. We therefore, transfected the EGR1 expression vector and examined osteosarcoma cell growth. Western blot analysis showed up-regulation of EGR1 in 143B, Saos-2, and HOS cells (**Figure 4A**). MTT assay revealed that forced expression of EGR1 did not prevent osteosarcoma growth in vitro (**Figure S3A**). We next examined the effects of EGR1 on anchorage-independent osteosarcoma growth. Colony formation assay revealed that forced expression of EGR1 did not affect the number of colony formation (**Figure S3B**). These findings suggest that up-regulation of EGR1 following anti-tumor agent treatment had no effect on osteosarcoma cell growth.

### Over-expression of EGR1 prevents osteosarcoma cell invasion in vitro

To examine the effects of EGR1 up-regulation after anti-tumour agent treatment in modulating the invasive activity of osteosarcoma cells, in vitro invasive activity assays were performed to assess the proportion of osteosarcoma cells transfected with EGR1 expression vector or control vector that invaded through matrigel-coated membranes. Significantly lower proportions of 143B, Saos-2, and HOS cells transiently transfected with EGR1 expression vector migrated through matrigel-coated chambers than osteosarcoma cells transfected with control vector (**Figure 4B, C**).

### Down-regulation of uPA and uPAR by EGR1

**Figure 5:**
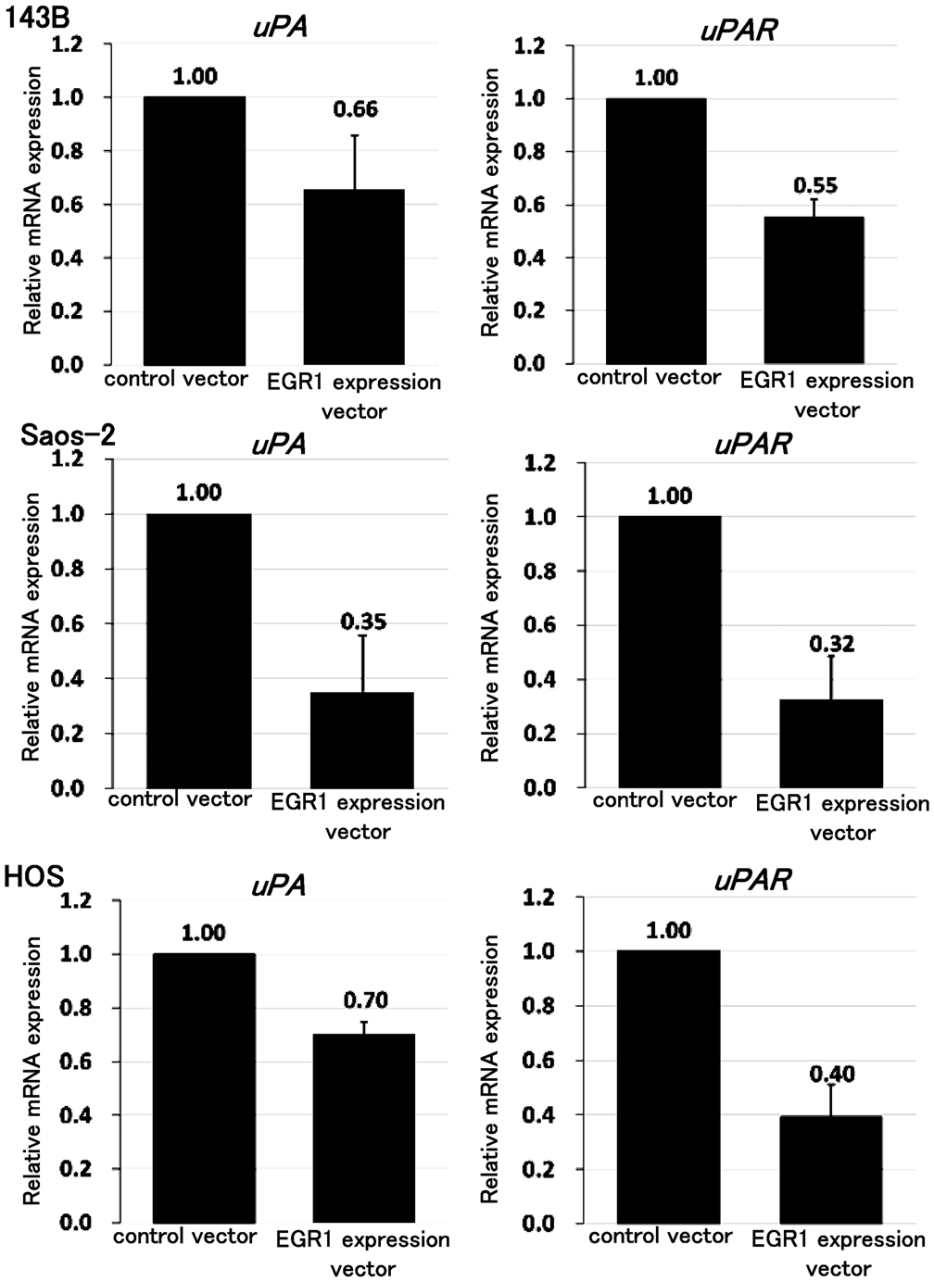
EGR1 decreased expression of *uPA* and *uPAR*. We examined whether EGR1 affects the expression of uPA and uPAR. RNA was prepared from control vector or EGR1 expression vector stably transfected cells. Real-time PCR revealed that forced expression of EGR1 decreased *uPA* and *uPAR* expression in 143B, Saos-2, and HOS cells (P<0.05). The comparative Ct (ΔΔCt) method was used to determine fold change in expression using *GAPDH*. These experiments were performed in triplicate with similar results [error bars represent mean (SD)].

We then examined the cellular mechanisms by which EGR1 exerts its effects on osteosarcoma cell invasion. Several investigations have shown that EGR1 plays an important role in the control of tumor metastasis through regulation of cancer invasion-related genes, including TGF-β1, thrombospondin-1, and plasminogen activator inhibitor-1 [17], [18]. We examined whether EGR1 affects the expression of cancer invasion-related genes. Real-time PCR revealed that forced expression of EGR1 in 143B, Saos-2, and HOS osteosarcoma cell lines decreased the expression of *uPA* and *uPAR* (**Figure 5**). ELISA revealed that forced expression of EGR1 decreased the expression of uPA and uPAR proteins (**Figure S4**). Further, we examine the effects of anti-tumour agents on *uPA and uPAR* expression in vitro, we performed real-time PCR after anti-tumor agent treatment. Treatment of low dose anti-tumor drugs decreased the expression of *uPA* and *uPAR* (**Figure S5**). To examine the effects of low dose chemotherapy on *uPA and uPAR* expression in vivo, we used osteosarcoma murine xenograft model with 143B cells. Nude mice were treated with 4 µg/kg doxorubicin. Real-time PCR showed that low dose doxrubicin treatment decreased expression of *uPA* and *uPAR* in vivo (**Figure S6A**).

### EGR1 down-regulates uPA activity

**Figure 6:**
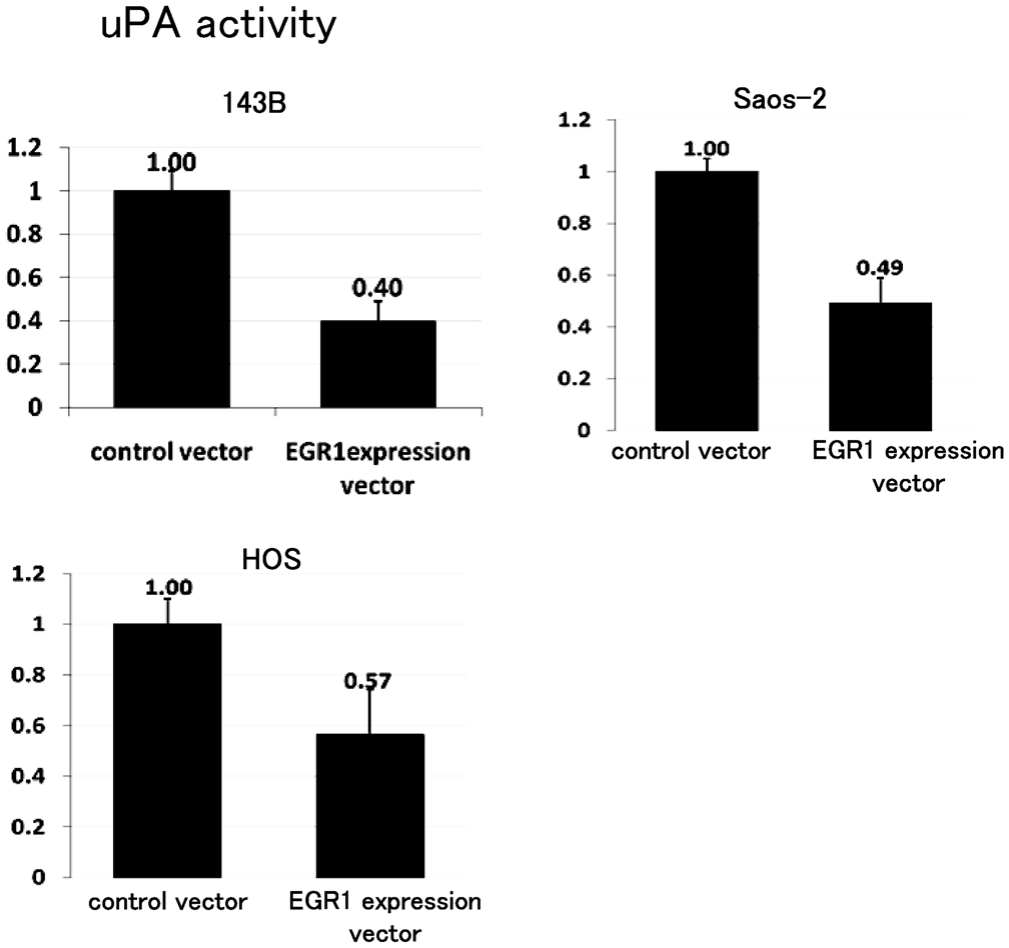
EGR1 decreased uPA activity. uPA activity was examined using cell lysates. Cell lysate were prepared from control vector or EGR1 expression vector transfected cells. ELISA assay showed that EGR1 decreased uPA activity 0.40-fold in 143B (A). uPA activity was decreased 0.49-fold by EGR1 in Saos-2 (B). Luciferase assay showed that EGR1 decreased uPA activity 0.57-fold in HOS (C). These experiments were in triplicate with similar results [error bars represent mean (SD)] (P<0.05).

uPA is produced and secreted as an inactive single-chain polypeptide, termed pro-uPA, which lacks plasminogen-activating activity. The binding of pro-uPA to uPAR induces its activation which in turn converts plasminogen to the active serine protease plasmin [19]. In this regard, we examined whether EGR1 exerts effects on uPA activity by performing uPA activity ELISA. ELISA showed that forced expression of EGR1 in osteosarcoma cell lines down-regulated the activity of uPA (**Figure 6**).

### EGR1 suppresses osteosarcoma migration into blood vessels in vivo

**Figure 7:**
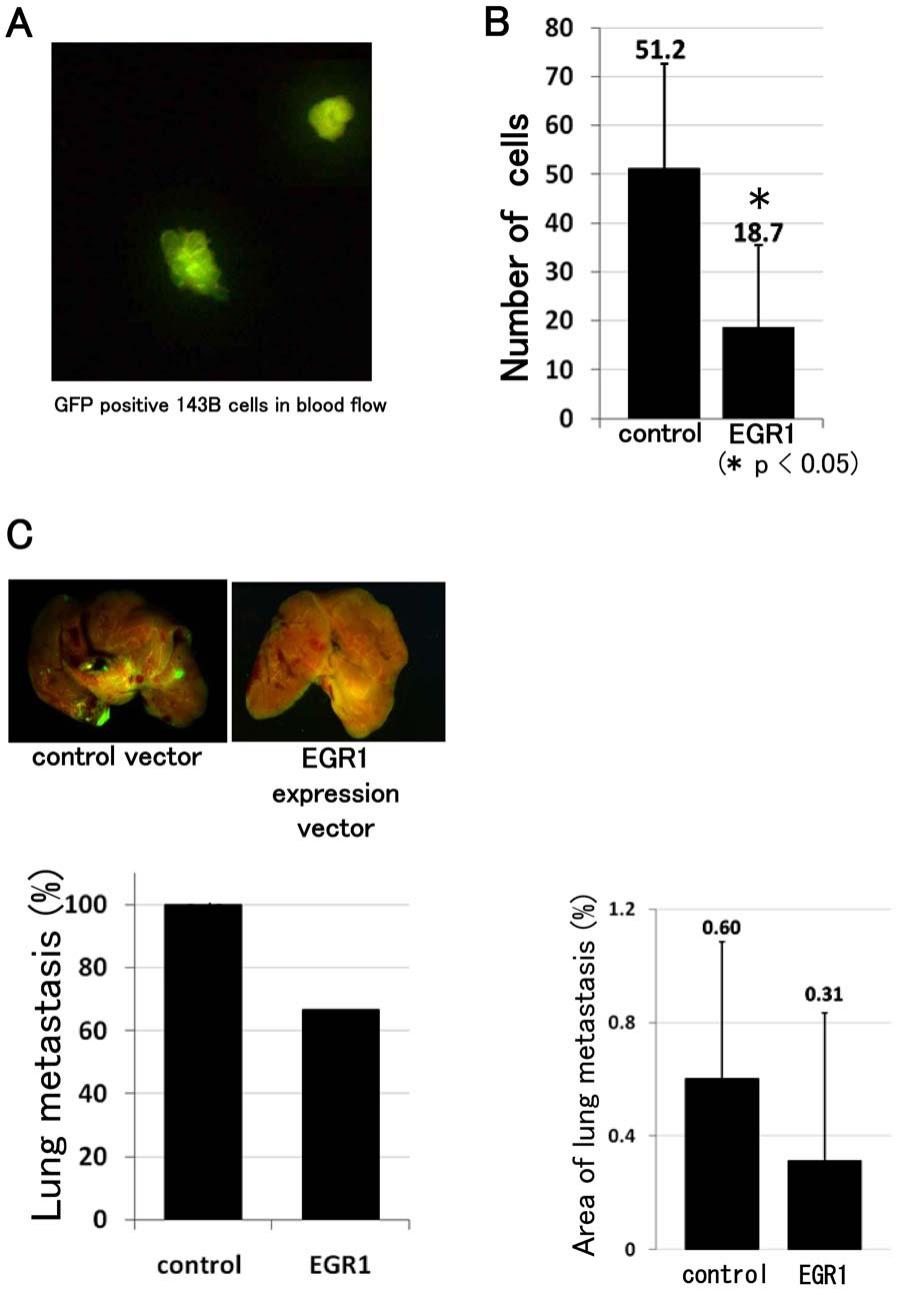
EGR1 prevents osteosarcoma cell migration into blood vessel in vivo. GFP expression virus-transfected 143B cells were inoculated into the knee joint. To examine tumour cell invasion of blood vessels, we counted GFP- positive- 143B cells within 50 µl blood aspirates from hearts at 5 weeks after inoculation (A). The number of GFP- positive cells in blood was decreased in EGR1 -expressing 143B- inoculated mice (B) [error bars represent mean (SD)] (P<0.05). Metastatic nodules in lungs were evaluated under fluorescence microscopy. Six of 6 control cell-inoculated mice exhibited lung metastases. Four of six (66.7%) EGR1-expressing cell-inoculated mice exhibited lung metastases. The percent of lung metastasis area was calculated by Lumina Vision (C).

To investigate the effects of EGR1 on osteosarcoma tumor migration and invasion in vivo, we used a novel osteosarcoma murine xenograft model with 143B cells. Intrajoint inoculation of GFP-positive 143B cells in nude mice induced primary osteosarcoma tumor formation by 2 weeks after inoculation. These primary tumors gave rise to microscopically detectible micro metastases in the lungs within 5 weeks after inoculation. Although we attempted to determine the volume of the primary tumors, we were unable to do so because tumor had extended into muscle and bone. RNA was prepared from tumor formed by control vector or EGR1 expression vector transfected cells. Real-time PCR revealed that forced expression of EGR1 decreased *uPA* and *uPAR* expression in vivo (**Figure S6B**). After 5 weeks, we counted GFP-positive- 143B cells within 50 µl blood aspirates from hearts. The vector control group had an average of 51.2 cells, whereas the EGR1 group averaged only 18.7 cells (**Figure 7A, B**). Lung metastases were found in 6 of 6 control cell-inoculated mice. In contrast, there were lung metastases in 4 of 6 EGR1-expressing 143B-inoculated mice. The percent of lung metastasis area was calculated. The vector control group had an average of 0.6% metastasis area, whereas the EGR1 group averaged 0.31% metastasis area (**Figure 7**C). These findings show that EGR1 prevented osteosarcoma migration into blood vessel in vivo.

## Discussion

Current standard regimens for osteosarcoma treatment include preoperative and postoperative chemotherapy. The benefits of chemotherapy have been demonstrated in many studies. Preoperative chemotherapy induces tumor necrosis in the primary tumor facilitating surgical resection and enabling early treatment of micrometastatic disease [20], [21]. Among those patients who received neoadjuvant treatment, chemotherapy-related tumor necrosis was good in 62% and poor in 38% of patients [22]. These findings showed that one-third of osteosarcoma patients are even poor responders to chemotherapy. Even though chemotherapy is quite benefit for osteosarcoma patients, administration of preoperative chemotherapy results in delay of surgical resection of primary tumor. It is possible that new lung metastases will develop during preoperative chemotherapy in poor or non-responders. We showed that low-dose anti-tumor agent treatment up-regulated EGR1 expression and that EGR1 prevented osteosarcoma invasion via uPA/uPAR down-regulation. These findings suggest that preoperative chemotherapy prevents the development of new lung metastases in poor or non-responders. In addition, osteosarcoma incidence rates in the United States peak in adolescence and in the elderly [23]. Many elderly patients cannot tolerate aggressive chemotherapy. Our findings suggest that low-dose chemotherapy might be useful for elderly osteosarcoma patients by preventing new metastasis when used in combination with radiation therapy or as maintenance therapy.

EGR1 has received much attention recently because of its wide range of activities as a transcription factor. Remarkably, EGR1 can exert effects as either a growth promoter or a tumor suppressor. EGR1 may induce or suppress cell proliferation or induce apoptosis of cancer cells [10], [16], [24]–[27]. Our MTT assay and colony formation assay showed that EGR1 over-expression had no effect on osteosarcoma cell growth. Our findings suggest that the expression of EGR does not have general effects on growth and instead exerts regulatory effects that appear to be cell-type-specific.

We showed up-regulation of EGR1 following cisplatin, methotrexate, etoposide, or doxorubicin treatment, each of which exerts cytotoxic effects by different pharmacological mechanisms. EGR1 can be rapidly induced by many stimuli, including growth factors, cytokines, ultraviolet light, anti-tumour agents, and various stresses [8,10,24, Cao, 1992 #88,25,28–35]. The distinct types of stress caused by anti-tumor drugs might promote up-regulation of EGR1, although anti-tumor drugs exert different pathways. Further, we examined which signaling pathway promotes EGR1 expression following anti-tumor agent treatment. We treated osteosarcoma cell lines with anti-tumor agent and some specific inhibitors including ERK inhibitor, HIF1-α inhibitor, JAK2 inhibitor, LY294002, and others but we were unable to inhibit EGR1 expression effectively. Further examination for regulation mechanisms of EGR1 expression is needed.

The principle mode of action of doxorubicin, an anthracycline antibiotic, appears to be its ability to cross-link DNA and RNA, thereby affecting DNA and RNA synthesis [36], [37]. However, recent studies have demonstrated that genotoxic (*i.e.*, DNA damaging) agents, including many important cancer chemotherapy drugs, can have significant and selective effects on the expression of certain inducible genes [38]. It has also been demonstrated that noncytotoxic doses of the DNA cross-linking cancer chemotherapy drugs MMC, cisplatin, and carboplatin were effective at significantly altering the expression of the *MDR1* gene coding for the multidrug resistance protein P-glycoprotein [37]. We were therefore interested in whether chemotherapy agents might similarly alter the expression of inducible invasion-related genes, and thereby potentially alter tumor invasiveness, and found that anti-tumour agents increased the expression of EGR1, and EGR1 decreased that of uPA and uPAR.

The uPA system is thought to play roles in several different processes important to tumor progression including angiogenesis, tumor growth, and metastasis [39]. Expression of uPA and uPAR frequently indicates a poor prognosis, and is in some cases predictive of invasion and metastasis. uPAR is also thought to play roles in the growth and metastasis of human osteosarcoma [40]–[44]. We showed that forced expression of EGR1 inhibited expression of uPA and uPAR. In addition, EGR1 decreased the activity of uPA. These findings suggest that up-regulation of EGR1 following chemotherapy inhibits osteosarcoma migration via uPA system. Many signaling pathways activate transcription factors that act on the uPAR promoter, driving uPAR expression in cancer [45]. uPAR transcription is controlled by ERK through activator protein 1 transcription factors [46]. Hypoxia-inducible factor 1α drive uPAR expression through a hypoxia responsive element in the uPAR promoter [47]. Nuclear factor-κB also activates uPAR expression [48]. Thus, multiple signaling inputs can up-regulate uPAR transcription in tumors. We could not detect the pathways that promote down-regulation of uPA/uPAR. Further examination for regulation mechanisms of uPA/uPAR system is needed.

Recently, many molecular target drugs have been developed [49]–[52]. In addition, several Notch signal inhibitors have been tested as molecular target drugs [53]–[55]. We previously reported that activation of Notch signaling promotes the progression of human osteosarcoma [56]. We examined the EGR1 expression by γ-secretase inhibitor, a pharmacological agent known to effectively block Notch activation. EGR1 was up-regulated by γ-secretase inhibitor in human osteosarcoma cell lines (data not shown). These findings suggest that EGR1 expression will also be up-regulated by molecular target drugs.

In summary, anti-tumor agents increased the expression of EGR1, and EGR1 decreased osteosarcoma invasion. Our findings suggest that even though chemotherapy could not prevent osteosarcoma growth in chemotherapy poor responders, chemotherapy prevents osteosarcoma cell migration into blood vessel by down-regulation of urokinase plasminogen activation via up-regulation of EGR1 during chemotherapy periods.

## Supporting Information

Figure S1 **Anti-tumor agent treatment increased the expression of** ***EGR1*** **.** Following 48 h or 5 days drug treatments, total RNA extracted from osteosarcoma cell lines were analyzed by real-time PCR. Following 48 h treatment, cisplatin, methotrexate, etoposide or doxorubicin increased *EGR1* expression in 143B cell and Saos-2 cells. Following 5 days treatment, cisplatin increased *EGR1* expression in 143B cell. Following 5 days treatment, etoposide or doxorubicin increased *EGR1* expression in Saos-2 cells. The comparative Ct (ΔΔCt) method was used to determine fold change in expression using *GAPDH* or *ACTB*. Experiments were performed in triplicate with similar results [error bars represent mean (SD)].
(TIF)

Figure S2 **Chemotherapy increased** ***EGR1*** **expression.** Total RNA extracted from osteosarcoma patients' biopsy specimens and excised tumors following chemotherapy were used for real-time PCR. Real-time PCR revealed that 3 of 3 excised specimens of osteosarcoma increased *EGR1* expression 7.87- to 1.73-fold (A). One day after 4 µg doxorubicin treatment, RNA was extracted from tumor in nude mice xenograft models. Real-time PCR revealed that low dose chemotherapy increased *EGR1* expression in vivo (B) (P<0.05). The comparative Ct (ΔΔCt) method was used to determine fold change in expression. These experiments were performed in triplicate with similar results [error bars represent mean (SD)].
(TIF)

Figure S3 **Forced expression of EGR1 does not affect osteosarcoma cell growth in vitro.** We transfected control vector or EGR1 expression vector, and examined osteosarcoma cell growth. MTT assay revealed that growth of viable 143B, Saos-2, and HOS cells over 8 days was not affected by forced expression of EGR1 (A). These experiments were performed in triplicate with similar results [error bars represent mean (SD)]. Colony formation assay revealed that forced expression of EGR1 did not affect the number of colonies in soft agar (B). These experiments were performed in triplicate with similar results [error bars represent mean (SD)].
(TIF)

Figure S4 **Forced expression of EGR1 decreased the expression of uPA and uPAR.** Cell lysate were prepared from control vector or EGR1 expression vector stably transfected cells. ELISA assay showed that forced expression of EGR1 decreased the expression of uPA and uPAR proteins in 143B (P<0.05) (A). The expression of uPA and uPAR decreased in Saos-2 and HOS (P<0.05) (B, C). These experiments were in triplicate with similar results [error bars represent mean (SD)].
(TIF)

Figure S5 **Low dose anti-tumor agent treatment decreased the expression of** ***uPA*** **and** ***uPAR*** **.** Following 24 h drug treatments, total RNA extracted from osteosarcoma cell lines were analyzed by real-time PCR. Treatment with cisplatin, methotrexate, etoposide or doxorubicin decreased *uPA and uPAR* expression in 143B and Saos-2 cells (P<0.05). The comparative Ct (ΔΔCt) method was used to determine fold change in expression using *GAPDH* or *ACTB*. Experiments were performed in triplicate with similar results [error bars represent mean (SD)].
(TIF)

Figure S6 **Chemotherapy prevents expression of** ***uPA*** **and** ***uPAR*** **by down-regulation of EGR1.** Twenty four hours after 4 µg doxorubicin treatment, RNA was extracted from tumour in nude mice xenograft model. Real-time PCR revealed that chemotherapy decreased *uPA* and *uPAR* expression in vivo (A) (P<0.05). To examined whether EGR1 affects the expression of uPA and uPAR in vivo. RNA was prepared from tumor formed by control vector or EGR1 expression vector transfected cells. Real-time PCR revealed that forced expression of EGR1 decreased *uPA* and uPAR expression in vivo (B) (P<0.05). The comparative Ct (ΔΔCt) method was used to determine fold change in expression using GAPDH. These experiments were performed in triplicate with similar results [error bars represent mean (SD)].
(TIF)
