# Supplementary figures and images for: Suppression of Osteosarcoma Cell Invasion by Chemotherapy Is Mediated by Urokinase Plasminogen Activator Activity via Up-Regulation of EGR1
Source: PLoS One. 2011 Jan 20;6(1):e16234. doi: 10.1371/journal.pone.0016234 (PMC3024416; doi:10.1371/journal.pone.0016234)

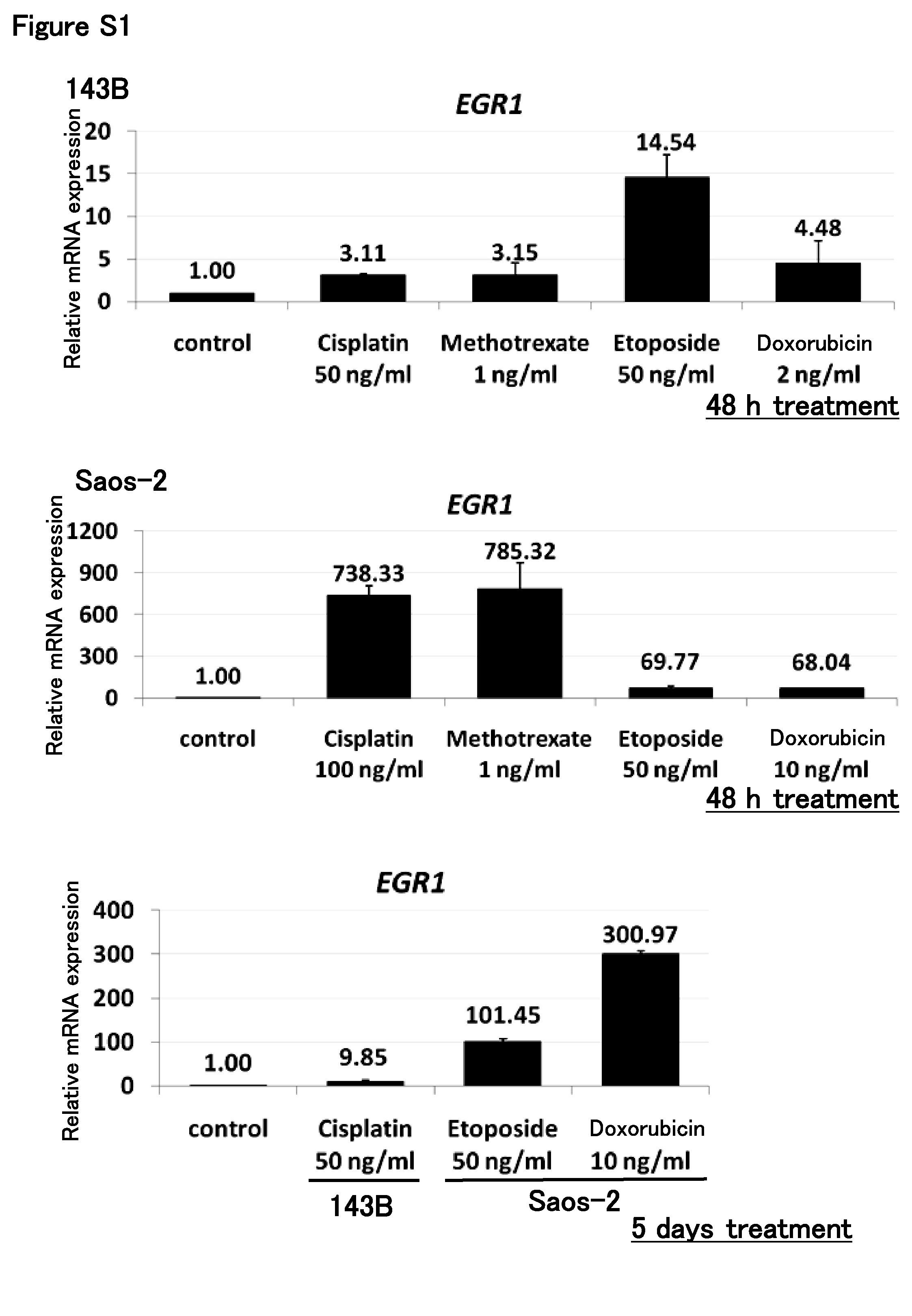

Supplement: Figure S1 — Anti-tumor agent treatment increased the expression of EGR1 . Following 48 h or 5 days drug treatments, total RNA extracted from osteosarcoma cell lines were analyzed by real-time PCR. Following 48 h treatment, cisplatin, methotrexate, etoposide or doxorubicin increased EGR1 expression in 143B cell and Saos-2 cells. Following 5 days treatment, cisplatin increased EGR1 expression in 143B cell. Following 5 days treatment, etoposide or doxorubicin increased EGR1 expression in Saos-2 cells. The comparative Ct (ΔΔCt) method was used to determine fold change in expression using GAPDH or ACTB. Experiments were performed in triplicate with similar results [error bars represent mean (SD)]. (TIF) [file pone.0016234.s001.tif]

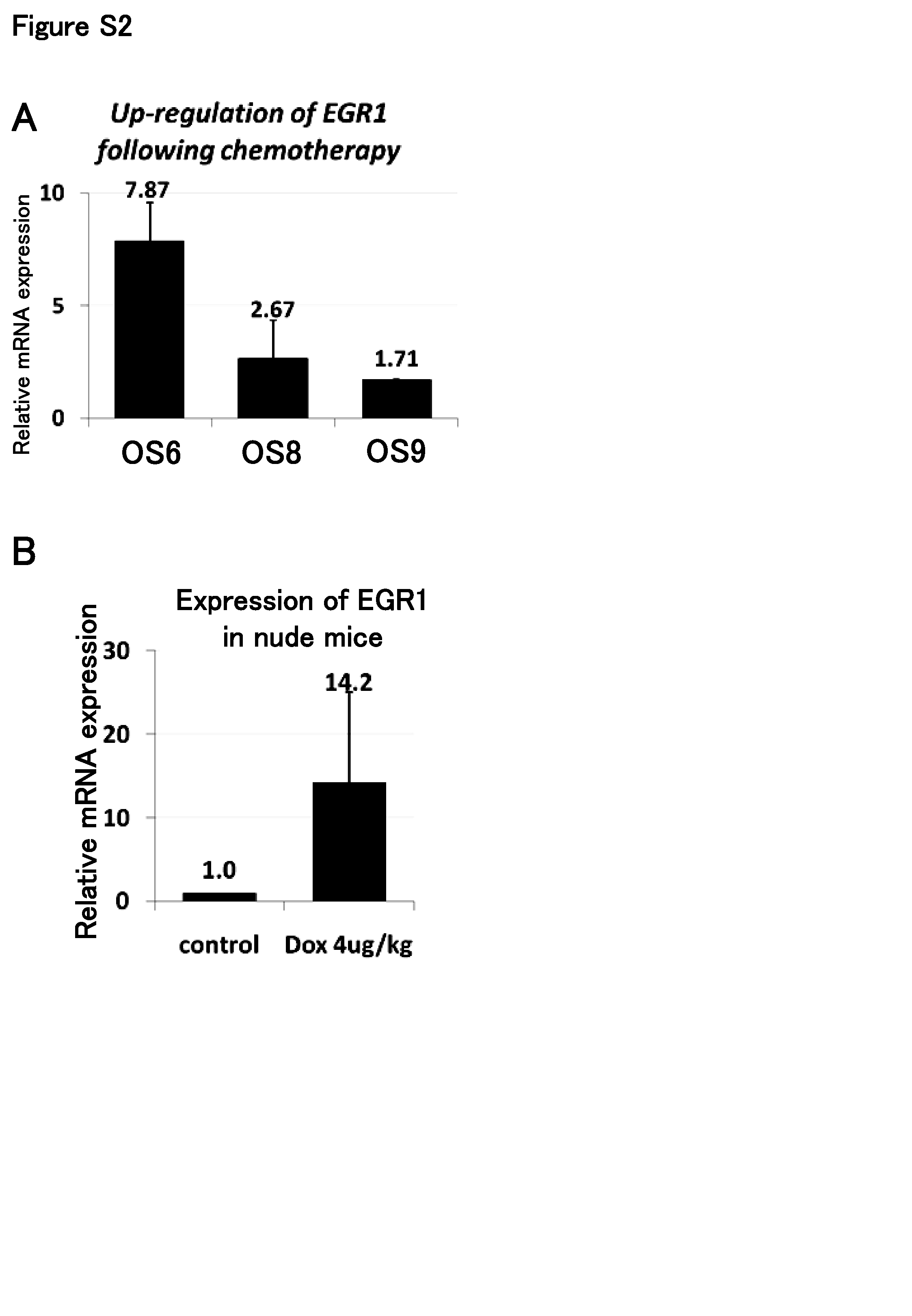

Supplement: Figure S2 — Chemotherapy increased EGR1 expression. Total RNA extracted from osteosarcoma patients' biopsy specimens and excised tumors following chemotherapy were used for real-time PCR. Real-time PCR revealed that 3 of 3 excised specimens of osteosarcoma increased EGR1 expression 7.87- to 1.73-fold (A). One day after 4 µg doxorubicin treatment, RNA was extracted from tumor in nude mice xenograft models. Real-time PCR revealed that low dose chemotherapy increased EGR1 expression in vivo (B) (P<0.05). The comparative Ct (ΔΔCt) method was used to determine fold change in expression. These experiments were performed in triplicate with similar results [error bars represent mean (SD)]. (TIF) [file pone.0016234.s002.tif]

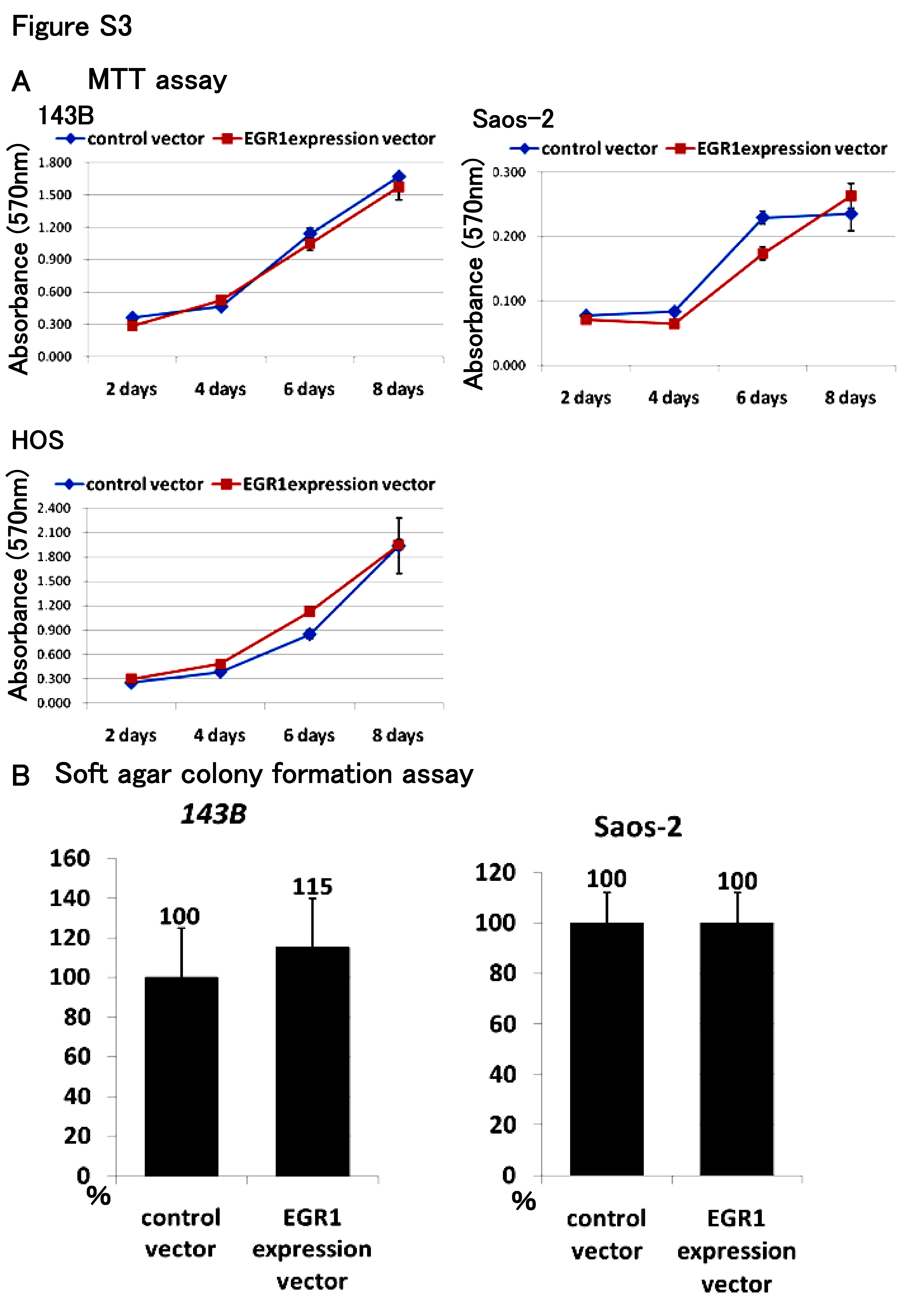

Supplement: Figure S3 — Forced expression of EGR1 does not affect osteosarcoma cell growth in vitro. We transfected control vector or EGR1 expression vector, and examined osteosarcoma cell growth. MTT assay revealed that growth of viable 143B, Saos-2, and HOS cells over 8 days was not affected by forced expression of EGR1 (A). These experiments were performed in triplicate with similar results [error bars represent mean (SD)]. Colony formation assay revealed that forced expression of EGR1 did not affect the number of colonies in soft agar (B). These experiments were performed in triplicate with similar results [error bars represent mean (SD)]. (TIF) [file pone.0016234.s003.tif]

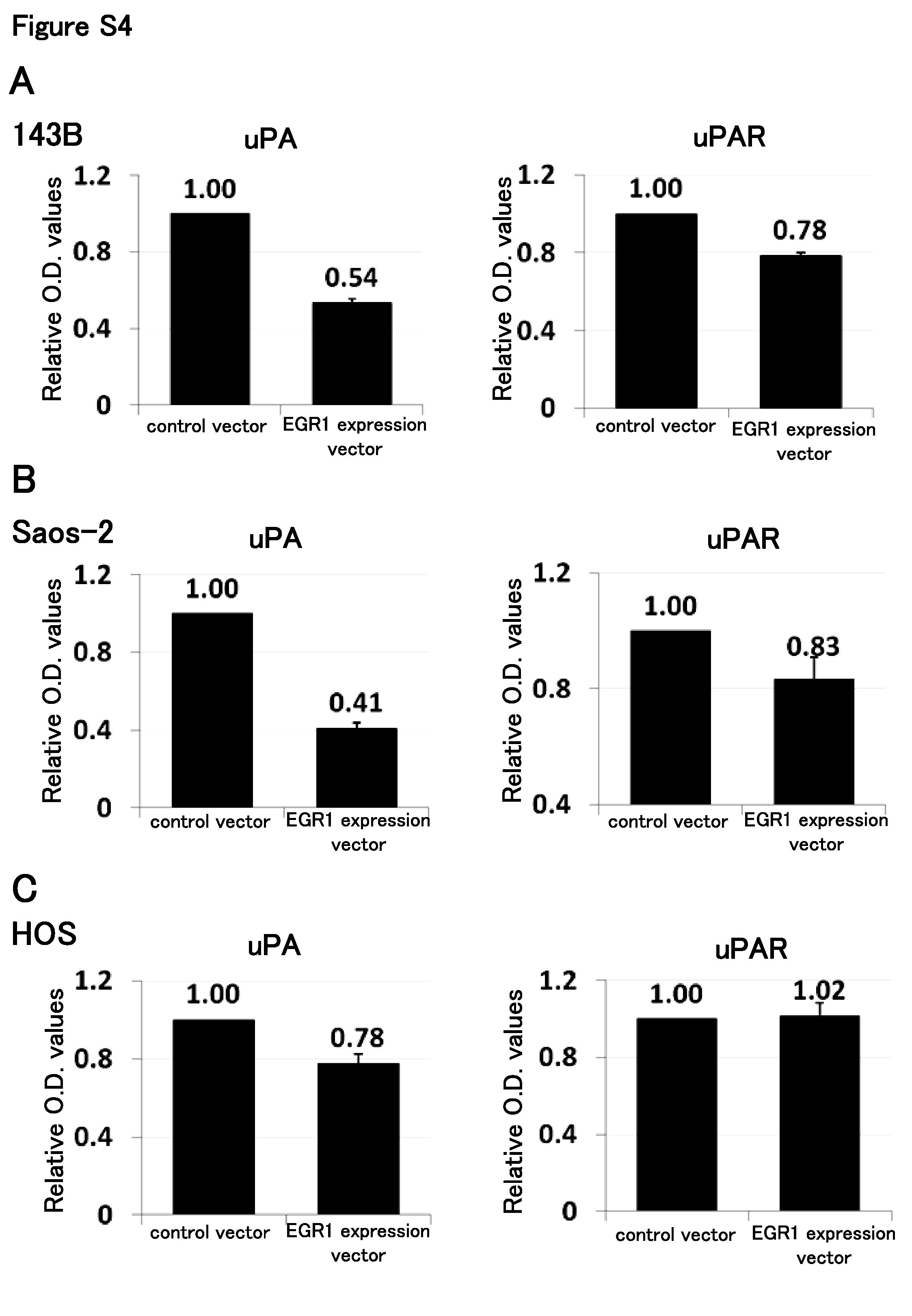

Supplement: Figure S4 — Forced expression of EGR1 decreased the expression of uPA and uPAR. Cell lysate were prepared from control vector or EGR1 expression vector stably transfected cells. ELISA assay showed that forced expression of EGR1 decreased the expression of uPA and uPAR proteins in 143B (P<0.05) (A). The expression of uPA and uPAR decreased in Saos-2 and HOS (P<0.05) (B, C). These experiments were in triplicate with similar results [error bars represent mean (SD)]. (TIF) [file pone.0016234.s004.tif]

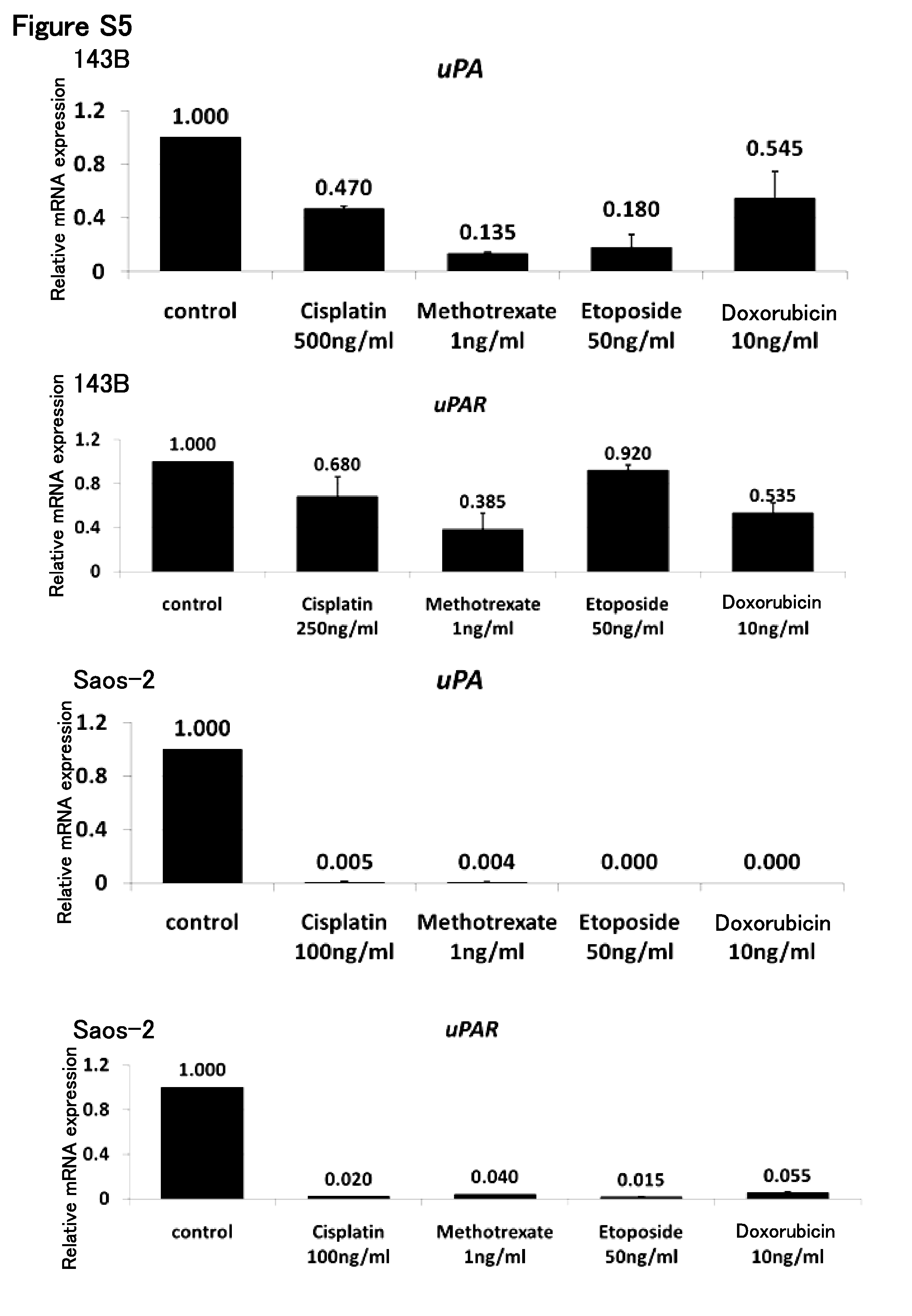

Supplement: Figure S5 — Low dose anti-tumor agent treatment decreased the expression of uPA and uPAR . Following 24 h drug treatments, total RNA extracted from osteosarcoma cell lines were analyzed by real-time PCR. Treatment with cisplatin, methotrexate, etoposide or doxorubicin decreased uPA and uPAR expression in 143B and Saos-2 cells (P<0.05). The comparative Ct (ΔΔCt) method was used to determine fold change in expression using GAPDH or ACTB. Experiments were performed in triplicate with similar results [error bars represent mean (SD)]. (TIF) [file pone.0016234.s005.tif]

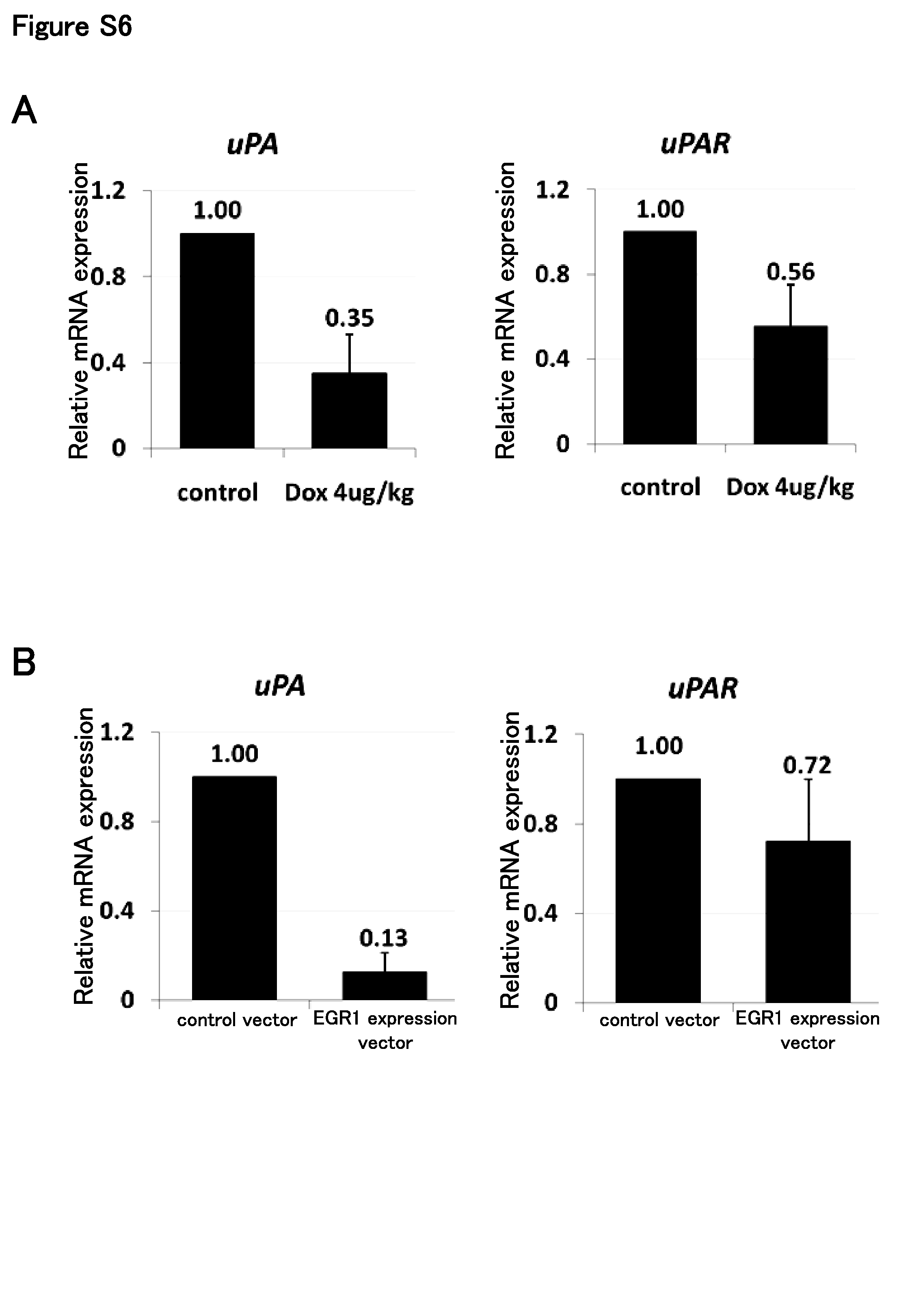

Supplement: Figure S6 — Chemotherapy prevents expression of uPA and uPAR by down-regulation of EGR1. Twenty four hours after 4 µg doxorubicin treatment, RNA was extracted from tumour in nude mice xenograft model. Real-time PCR revealed that chemotherapy decreased uPA and uPAR expression in vivo (A) (P<0.05). To examined whether EGR1 affects the expression of uPA and uPAR in vivo. RNA was prepared from tumor formed by control vector or EGR1 expression vector transfected cells. Real-time PCR revealed that forced expression of EGR1 decreased uPA and uPAR expression in vivo (B) (P<0.05). The comparative Ct (ΔΔCt) method was used to determine fold change in expression using GAPDH. These experiments were performed in triplicate with similar results [error bars represent mean (SD)]. (TIF) [file pone.0016234.s006.tif]
